# Pattern of Fine Needle Aspiration Cytology of Head and Neck Swelling in Patients Attending a Tertiary Health Care Center: A Descriptive Cross-sectional Study

**DOI:** 10.31729/jnma.8714

**Published:** 2024-08-31

**Authors:** Soorya Bhattarai, Sapana Sedhain, Neeta Kafle, Amrita Sinha

**Affiliations:** 1 Department of Pathology, Birat Medical College Teaching Hospital, Tankisinuwari, Morang, Nepal

**Keywords:** *fine needle aspiration*, *head and neck neoplasm*, *lymph nodes*

## Abstract

**Introduction:**

Fine needle aspiration cytology is a simple, rapid, cost-effective method in diagnosis of head and neck swelling with minimal risk of complications. Head and neck swellings include a broad spectrum of diseases with different management for each. Fine needle aspiration cytology is a suitable and useful method for assessment of these swelling. This study was done with the objective to study the frequency and distribution of various head and neck lesions detected by fine needle aspiration cytology.

**Methods:**

A descriptive cross-sectional study was conducted at the Department of Pathology in a tertiary care center from February 1 to July 31, 2023 after obtaining ethical approval from Institutional Review Committee (Reference number: IRC-PA-191/2078-79). All the patients presenting with head and neck swelling during the study period were included in this study. Total sampling was done. Fine needle aspiration was done and cytological diagnosis was made. Descriptive analysis was done where frequency and percentage were calculated.

**Results:**

Out of 112 cases included in the study, 43 (38.40%) were of lymph nodes, 36 (32.14%) of thyroid, 22 (19.64%) of skin and soft tissue and 11 (9.82%) of salivary glands. Among the lymph nodes cases, there were 11 (25.57%) metastases. In thyroid lesions, beingn lesions were seen in 24 (66.68%).

**Conclusions:**

This study found that lymph nodes were the most common site for head and neck swellings, frequently involving metastatic lesions.

## INTRODUCTION

Head and neck swelling is a commonly encountered clinical problem and sometimes present as incidental lump with malignancy.^1,2^ The diagnosis of these lesions are apparent from sites and clinical features but can be confusing sometimes.^3^ Head and neck region has multiple accessible organs like thyroid, salivary glands and heterogeneous pathology of soft tissue and lymph nodes along with distance metastasis and recurrences.^4^

Fine needle aspiration cytology (FNAC) is a simple, rapid and cost effective method in diagnosing swellings with minimal risk of complications.^5,6^ The test can be performed as an outpatient procedure.^7^ Though FNAC cannot give architectural detail, it can be representative of entire lesion through aspirate.^8^

FNAC is beneficial in early differentiation of benign lesions from malignant, influencing its management.^5^

Since head and neck swellings include a broad spectrum of diseases, this study was done to study the frequency and distribution of various head and neck lesions detected by FNAC.

## METHODS

This study was a descriptive cross-sectional study conducted from February 1 to July 31, 2023 at the Department of Pathology of tertiary care center of eastern Nepal. The data was collected after the ethical approval from the Institutional Review Committee (Reference number: IRC-PA-191/2078-79). Patients of all age groups and both sexes with palpable head and neck swelling presenting to the department of pathology for FNAC during the study period of six months were included in the study. After taking informed consent and detailed history, the lump was assessed. Total sampling was done where all the patients during the study period were included. Hence, 112 were included.

Under aseptic conditions a syringe with a 22-gauge needle was inserted into the swelling. Ultrasound assistance was taken whenever needed. After positioning into target tissue, the needle was moved back and forth and material was aspirated, the needle was withdrawn. Air dried and wet fixed smear (95% alcohol) was prepared and stained with Giemsa and Papanicolau stains respectively. Special stains (like Zeihl Neelssen) were done whenever required. The smears were observed under microscope and interpreted as per need and then entered in preformed proforma.

The data were entered in Microsoft Excel and analyzed using Statistical Package for the Social Sciences (IBM SPSS). Descriptive analysis was done where frequency and percentage were calculated.

## RESULTS

Our study included a total of 112 patients, comprising of 74 female (66.07%) and 38 male (33.93%) patients. Among samples, 43 (38.40%) were from lymph nodes and 36 (32.14%) from thyroid (Table 1).

Age of the patients ranged from 1 to 81 years with mean age being 40.29±19.48 years. The one year old male child had suppurative lesion (lymph node) while 81 year old male had metastatic squamous cell carcinoma (lymph node) in FNAC.

**Table 1:** Distribution of lesions as per sites involved (n = 112).

Lesion	Female n (%)	Male n (%)	Total n (%)
Thyroid	32 (28.57)	4 (3.57)	36 (32.14)
Lymph node	20 (17.86)	23 (20.54)	43 (38.40)
Skin and soft tissue	14 (12.50)	8 (7.14)	22 (19.64)
Salivary glands	8 (7.14)	3 (2.68)	11 (9.82)
**Total**	**74 (66.07)**	**38 (33.93)**	**112 (100)**

Lymph node metastases comprised 11 (25.57%) cases out of which 6 (54.54%) were squamous cell carcinoma and 1 (9.09%) adenocarcinoma, nasopharyngeal carcinoma and melanoma each. Remaining 2 (18.18%) were not further classified but only designated as metastases (Table 2).

**Table 2:** Distribution of various lymph node lesions (n = 43).

Lesions	Female n (%)	Male n (%)	Total n (%)
Metastases	4 (9.30)	7 (16.27)	11(25.57)
Reactive lymphadenitis	4 (9.30)	4 (9.30)	8 (18.60)
Tuberculosis	4 (9.30)	4 (9.30)	8 (18.60)
Lympho proliferative disorder	4 (9.30)	3 (6.98)	7 (16.28)
Granulomatous lymphadenitis	3 (6.98)	3 (6.98)	6 (13.96)
Suppurative lesion	1 (2.33)	1 (2.33)	2 (4.66)
Kikuchi disease	-	1 (2.33)	1 (2.33)
**Total**	**22 (46.51)**	**21(53.49)**	**43 (100)**

There were 36 (32.14%) cases of thyroid lesions out of which 24 (66.68%) were benign (category II) and of which 8 (33.33%) were composed of lymphocytic thyroiditis and 16 (66.67%) cases of the benign follicular nodule. Benign follicular nodule included nodular goitre 12 (33.34%) and colloid nodule 4 (9.30%) cases. The benign category was followed by malignancy (category VI) comprising 4 (11.11%) cases. All the four cases of malignancy were further classified as papillary thyroid carcinoma (Table 3).

**Table 3:** Distribution of thyroid gland lesions (according to Bethesda system for reporting thyroid cytopathology)^9^ (n = 36).

Category according to Bethesda system	Male n (%)	Female n (%)	Total n (%)
Category I (non-diagnostic)	-	2 (5.55)	2 (5.55)
Category II (benign)	3 (8.33)	21 (58.35)	24 (66.68)
Category III (atypia of undetermined significance)	1 (2.78)	2 (5.55)	3 (8.33)
Category IV (follicular neoplasm)	-	1 (2.78)	1 (2.78)
Category V (suspicious for malignancy)	-	2 (5.55)	2 (5.55)
Category VI (malignant)	-	4 (11.11)	4 (11.11)
**Total**	**4 (11.11)**	**32 (88.89)**	**36 (100)**

The lipoma comprised 8 (36.36%) cases and benign cystic lesions comprised 6 (27.27%) (Table 4).

**Table 4:** Distribution of skin and soft tissue lesions (n = 22).

Lesion	Male n (%)	Female n (%)	Total n (%)
Lipoma	2 (9.09)	6 (27.27)	8 (36.36)
Benign cystic lesion	2 (9.09)	4 (18.18)	6 (27.27)
Epidermal inclusion cyst	2 (9.09)	3 (13.64)	5 (22.73)
Unsatisfactory	2 (9.09)	-	2 (9.09)
Schwannoma	-	1 (4.55)	1 (4.55)
**Total**	**8 (36.36)**	**14 (63.64)**	**22 (100)**

Among salivary glands pathology, there were 3 cases (27.27%) non neoplastic consisting of sialadenitis and benign neoplasm consisting of pleomorphic adenoma. 2 cases (18.2%) were malignant with a differential of mucoepidermoid carcinoma and squamous cell carcinoma.

**Table 5:** Distribution of salivary gland lesions: Milan system for reporting salivary gland cytopathology.^10^ (n = 11).

Category according to Milan system	Male n (%)	Female n (%)	Total n (%)
Category I (non-diagnostic)	-	-	-
Category II (non-neoplastic)	1 (9.09)	2 (18.18)	3 (27.27)
Category III (atypia of undetermined significance)	-	1 (9.09)	1 (9.09)
Category IV A neoplasm: benign	1 (9.09)	2 (18.18)	3 (27.27)
Category IV B neoplasm: salivary gland neoplasm of uncertain malignant potential	-	2 (18.18)	2 (18.18)
Category V (suspicious of malignancy)	-	-	-
Category VI: malignant	1 (9.09)	1 (9.09)	2 (18.18)
**Total**	**3 (27.27)**	**8 (72.73)**	**11 (100)**

## DISCUSSION

Our study showed lymph node swellings were highest in frequency which was followed by thyroid lesions which was similar to the study done in various parts of India and Nepal.^2,11^'^13^ In these studies the total number of cases ranges from 100 (Sangavi) to 300 (Shreedevi).^2,11^ In study done by Sangavi et al, Kaur et al and Shreedevi lymph node comprises of 41%, 52.89% and 50.32% respectively which was followed by thyroid (37%, 33.47% and 44.07% respectively).^2,11,12^

Similarly, in study done by Shrestha et al., out of 154 cases, lymph nodes comprised of 58% which was followed by thyroid (23%).^13^ Largest number of aspirates in the study done by Padia et al., were from lymph nodes (64.02%) among 139 patients which was followed by thyroid (18.7%), skin and soft tissue (12.94%) and salivary glands (2.87%).^1^ Salivary gland pathology was the least common in our study (9.8%) similar to other studies. In a study done by Shrestha et al. and Shreedevi et al., salivary glands comprised of 5% and 3.28% respectively.^11,13^

In our study, metastatic deposits were predominant findings in lymph nodes in contrast to study done by Padia et al. and Shreedevi et al., for one year duration which showed reactive lymphadenitis as predominant findings.^1,11^ Metastasis 11 (25.57%) which in combined with lymphoproliferative disorder comprised nearly 50% of the malignant cases in our study. Metastatic deposits were most commonly seen in the patients over 50 years of age in our study. Out of 43 cases of lymph nodes, 14 (32.56%) cases in our study were above 50 years of age. The most common metastases encountered was squamous cell carcinoma in our study. In study done by Modi et al., which Metastatic squamous cells comprises of 28.57% and 3.74% of lymph nodes pathologies.^5,14^ Increase in malignancies could be due to the fact that our institute is a tertiary oncology center and there is rising awareness among patients in regards to need for further examination of swellings. The cases diagnosed as lymphoproliferative disorders were further advised for histopathological examination for further confirmation and categorization. Shrestha S. in their study showed more than 50% of cases comprising of inflammatory or infectious origin (tubercular, suppurative and granulomatous) which comprises 37% in our study.^13^ The diagnosis of tubercular lymphadenitis was made in our study only when either acid fast bacilli and/or caseous necrosis with granuloma was seen in the smears. This may be the cause of low incidence of tuberculosis seen in our study as those cases with granuloma but negative acid fast bacilli were classified as separate entities granulomatous lymphadenitis.

The FNAC of thyroid were classified as per Bethesda system of reporting thyroid cytopathology in our study.^9^ Among 36 cases of thyroid in our study, 2 (5.55%) were diagnosed as non'diagnostic due to suboptimal cellularity. Category II included benign conditions like nodular goiter, lymphocytic thyroiditis and colloid nodule comprising 66.68% of the cases with 3 males and 21 females. In a study done by Patel et al, out of 57 cases of thyroid, 84% cases were benign.^15^ Category V and VI comprised 5.55% and 11.11% cases respectively. The patients under these two categories were all females. All Category V cases were further categorized as suspicious of papillary thyroid carcinoma and category VI cases as papillary thyroid carcinoma. These findings are similar to study done by Shrestha et al.^13^

According to our study the most common skin and soft tissue tumors of head and neck region was lipoma (36.36%) in contrast to Jadhav in which epidermal inclusion cyst was commonest.^6^ The most common site of lipoma was nape of the neck. Lipoma was followed by benign cystic lesion in our study. These swelling (benign cystic lesions) subsided or decreased in size on aspiration. In 2 cases, no diagnosis could be reached in our study as they yielded only bloody aspirate and labeled as unsatisfactory in our study. There were no any malignant lesions in skin and soft tissue similar to study done by Modi et al.^14^

The salivary glands FNAC were further classified according to Milan system.^10^ The common lesions were non-neoplastic and benign neoplasm (27.27%) similar to Padia et al.^1^ In non-neoplastic group all three cases were classified as sialadenitis and among benign neoplasm all three cases were diagnosed as pleomorphic adenoma in our study. Two cases were categorized as malignant with a differential of high grade mucoepidermoid carcinoma and squamous cell carcinoma in our study. These two malignant cases were detected in parotid glands.

Majority of the patients in our study were female, similar to other studies done by Solanki et al. and Prabhakar et al., where male to female ratio ranges from 1:1.5 to 1:2.^16,17^ In the present study, the age ranges from 0-81 years. This is comparable to the study conducted by Padia et al.^1^ FNAC is used to obtain the sample of the cells from the suspicious mass for diagnostic purposes. Hence, FNAC is widely used these days in head and neck lesions as it aims to provide clinically useful information that exceeds the results obtained by palpation and imaging alone.^1^

FNAC lacks histological typing and gives information regarding cytological architecture only. Accuracy of FNAC depends upon the location, pathologic type of mass, expertise and sample adequacy. Hence our study has limitations in sub-classifying various lymphoproliferative disorders and classifying different types of reactive lymphadenitis where histopathological correlation is warranted. Besides, follow-up for unsatisfactory or non-diagnostic samples were not done in our study.

## CONCLUSIONS

In our study, lymph node pathology was more commonly encountered head and neck swelling which is followed by thyroid. FNAC can further help in classifying the nature of these swellings (inflammatory, benign and malignant) which further aids in management of the patients.
